# The Recent Applications of Magnetic Nanoparticles in Biomedical Fields

**DOI:** 10.3390/ma17122870

**Published:** 2024-06-12

**Authors:** Jiaqi Hong, Linhao Wang, Qikai Zheng, Changyu Cai, Xiaohua Yang, Zhenlin Liao

**Affiliations:** College of Food Science, South China Agricultural University, Guangzhou 510642, China; jiaqi@stu.scau.edu.cn (J.H.); 20213164064@stu.scau.edu.cn (L.W.); zqk15897671790@163.com (Q.Z.); caichangyu@stu.scau.edu.cn (C.C.); y-xiaohua@stu.scau.edu.cn (X.Y.)

**Keywords:** magnetic nanoparticles, biomedicine, detection, imaging, treatment

## Abstract

Magnetic nanoparticles (MNPs) have found extensive application in the biomedical domain due to their enhanced biocompatibility, minimal toxicity, and strong magnetic responsiveness. MNPs exhibit great potential as nanomaterials in various biomedical applications, including disease detection and cancer therapy. Typically, MNPs consist of a magnetic core surrounded by surface modification coatings, such as inorganic materials, organic molecules, and polymers, forming a nucleoshell structure that mitigates nanoparticle agglomeration and enhances targeting capabilities. Consequently, MNPs exhibit magnetic responsiveness in vivo for transportation and therapeutic effects, such as enhancing medical imaging resolution and localized heating at the site of injury. MNPs are utilized for specimen purification through targeted binding and magnetic separation in vitro, thereby optimizing efficiency and expediting the process. This review delves into the distinctive functional characteristics of MNPs as well as the diverse bioactive molecules employed in their surface coatings and their corresponding functionalities. Additionally, the advancement of MNPs in various applications is outlined. Additionally, we discuss the advancements of magnetic nanoparticles in medical imaging, disease treatment, and in vitro assays, and we anticipate the future development prospects and obstacles in this field. The objective is to furnish readers with a thorough comprehension of the recent practical utilization of MNPs in biomedical disciplines.

## 1. Introduction

**Figure 1:**
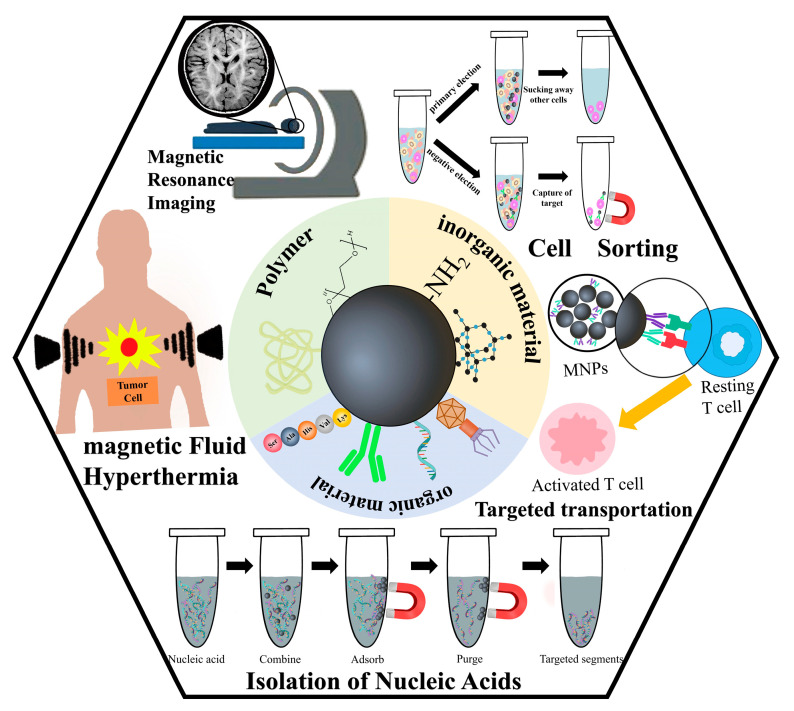
Schematic illustration of the main applications of magnetic nanoparticles (MNPs) with functional surface coatings.

When compared to other nanostructures, magnetic nanoparticles (MNPs) are one of the most significant and often employed classes of nanomaterials because of their specific characteristics. MNPs possess the capacity to significantly impact diagnostic and therapeutic practices in the medical field due to their distinctive characteristics, such as super-paramagnetic moment, magnetic resonance, and efficient interactions at the molecular and cellular levels [1,2]. Additionally, the application of diverse surface coatings can enhance the medicinal properties of MNPs, leading to desirable pharmacokinetic effects and reduced toxicity. Apart from properties, attractive and durable medicinal properties can be produced for these particles by using various surface coatings, and the pharmacokinetic effects and toxicity of magnetic nanoparticles caused by interactions with cells or biological proteins can be avoided, resulting in the increased biocompatibility and practicality of magnetic nanoparticles [3,4]. MNPs are used in a wide variety of fields, especially in biomedical applications [5] (Figure 1). MNPs have demonstrated utility as a medical imaging contrast agent [6,7,8], as a carrier for pharmaceutical agents [9,10], and in cancer therapy through magnetically induced hyperthermia (MHT) [11,12,13], photodynamic therapy (PDT) [6,14,15], and photothermal therapy (PTT) [16,17,18].

On the other hand, MNPs have garnered significant interest as valuable tools for in vitro diagnosis with a variety of appealing applications such as trace analysis, magnetic separation, quantitative detection, and rapid testing, making them highly suitable for a range of biomedical uses [19]. Furthermore, MNPs serve as safe and efficient vectors for gene delivery, facilitating various biotechnological techniques including magnetofection [20], molecular recognition systems [21], single-nucleotide polymorphism (SNP) genotyping [22,23], and gene-specific PCR assays [24,25].

Therefore, MNPs are extensively researched due to their diverse applications. This paper aims to delve into the characteristics of MNPs, including their magnetic properties, size, and the bioactive molecules incorporated in the shell layer. Additionally, it discusses the advancements in utilizing MNPs for medical imaging, disease treatment, and in vitro assays as well as the potential future directions and obstacles in the development of MNPs. The core–shell structure of MNPs and their functions are comprehensively introduced in this study. We present a comprehensive view of MNPs from the inside out, from the core–shell structure of MNPs, the roles of cores and coatings, to the applications of mature products, which include various landed MNP products, and MNP clinical applications approved for use in various countries, with the aim of providing readers with a comprehensive understanding of the practical applications of magnetic nanoparticles in biomedical fields. The objective is to furnish readers with a thorough comprehension of the practical utilization of MNPs in biomedical disciplines.

## 2. Characteristics of MNPs

### 2.1. Functional Characteristics of MNPs

Distinguished from conventional nanomaterials, MNPs may exhibit distinctive properties: namely, superparamagnetism, magnetic guidance, high transverse relaxivity, magnetothermal effects and other magnetic properties [26]. When smaller than the critical size, for temperatures below the Curie temperature but above the blocking temperature, MNPs will be in a superparamagnetic state. Superparamagnetic MNPs are highly magnetizable under an applied external magnetic field, typically presenting high magnetic susceptibility and saturation magnetizations but with no magnetic coercivity and hence no remanent magnetization. This is particularly relevant since after removing the applied magnetic field, the nanoparticles will exhibit close to null magnetic-related agglomeration, hence enabling favorable dispersions. This superparamagnetic behavior facilitates targeting and magnetic separation in various product applications. Magnetic fluid hyperthermia (MFH) relies on MNPs to convert energy of alternating magnetic fields (AMF) into thermal energy. The MNPs located in tumors and other diseased areas generate localized heating under the application of AMF, inducing selective apoptosis or necrosis of the diseased cells [27]. The transverse relaxivity of MNPs induces the spin relaxation of water protons when imaging tissues in vivo, increasing the T_1_ (spin–lattice) and T_2_ (spin–spin) relaxivity of water protons, thereby further enhancing magnetic resonance imaging (MRI) contrast [28]. Notably, MNPs exhibit antimicrobial properties to a certain extent [29]. Some authors believe that electrostatic forces can cause NPs to bind to the bacterial cell membrane and disorganize their metabolic functionality, while others propose that the production of reactive oxygen species (ROS) is the primary bactericidal mechanism utilized by numerous antibacterial NP agents [30].

### 2.2. Biocompatibility of MNPs

MNPs utilized in various application scenarios have distinct requirements for desirable product characteristics. In vivo applications necessitate MNPs with optimal biocompatibility and particle size to prevent aggregation resulting from magnetic attraction, thereby ensuring stability. Additionally, it is crucial for MNPs to possess the ability to evade recognition and capture by the reticuloendothelial system, traverse the capillary network without inducing thrombosis, and enhance the likelihood of reaching target tissues [31]. As such, the size and surface coating of MNPs are pivotal factors influencing their non-toxicity and chemical properties. The utilization of biocompatible materials as coatings for magnetic nanoparticles can serve as a protective barrier, improving the overall biocompatibility of the particles by facilitating the continuous adaptation of more biocompatible surface coatings. This process can help prevent rejection, cytotoxicity, and carcinogenicity in vivo [32].

Within the tissue body, nanoparticles larger than 200 nm in diameter are readily cleared by the reticuloendothelial system [30]. However, nanoparticles with diameters smaller than 8 nm are easily excreted from the body through existent pores of the kidney’s basal lamina (renal clearance) [33], reducing the blood-circulating time of these nanostructures. The diameter range of 10–40 nm (including ultrasmall MNPs) is fundamental for prolonged blood circulation, allowing the nanoparticles to cross capillary walls and often be phagocytized by macrophages trafficking to the lymph nodes and bone marrow. MNPs sized between 50 and 100 nm have the ability to evade the reticuloendothelial system in vivo, resulting in extended circulation periods and circumventing clearance by alveolar macrophages [34]. Therefore, optimizing the size of MNPs so that they avoid pharmacokinetic effects and toxicity resulting from interactions with cellular or biological proteins, as well as overcoming in vivo barriers, can enhance the effective surface area (primarily applicable to particles with sizes below 100 nm) and improve tissue diffusion [35,36].

From the above, it can be seen that MNPs, as a unique presence in nanomaterials, possess irreplaceable magnetic properties and are widely favored as magnetic targeting carriers. As a result, MNPs are synthesized with different strategies focusing on different internal magnetic cores, sizes, and surface-modified layers are usually used to constitute MNPs to meet specific requirements. Furthermore, MNPs for medical imaging require high transverse relaxation and resolution, MNPs for hyperthermia are critical for heat efficiency, and targeted delivery requires high targeting precision and specificity. Magnetic nanoparticle products for in vitro applications are capable of high throughput, high sensitivity, and automated operation.

## 3. Functional Surface Coatings on MNPs

The agglomeration of metal oxides occurs, which seriously affects the stability and dispersion of the particles, thereby restricting the functionality of MNPs [37]. Furthermore, MNPs utilized in biological settings typically necessitate a protective coating to mitigate the detrimental effects of dissolved oxygen, ROS, and bioactive compounds. As a result, a compact and chemically stable shell grows on the core around the metal oxide. Common coating materials are categorized into inorganic materials, organic molecules, and polymers [38].

Functional surface coatings offer a range of possibilities for MNPs. Firstly, these coatings provide limited zwitterionic properties to enhance hydrophobicity and dispersion while safeguarding the magnetic core from damage and erosion [30]. Secondly, coatings act as a barrier, effectively shielding the magnetic core against the attack of chemical species in the aqueous solution and preventing rejection by the body. which completely improves the biocompatibility and optimizes the pharmacokinetics of the body via escaping the rapid elimination “stealth effect” [39]. Certain surface coatings impart specific recognition functions to MNPs in order to regulate the timing of drug release or improve the accuracy of binding to targeting units [40], which makes the delivery of MNPs in vivo more maneuverable and accurate [41].

### 3.1. Inorganic Material

Inorganic materials, specifically carbon, noble metals, and inert oxides like gold [42], silver [43], silica [44], and alumina [45], have been utilized in the coating of MNPs to improve magnetism stability. The decoration of MNPs with noble metals (gold or silver) enables new properties such as optical properties and enhanced bio-affinity, biocompatibility, and chemical and physical properties without affecting the magnetic features of the core. Thus, noble metal MNPs are extensively employed for surface modification of the core in various applications, such as electrochemical (bio)sensing, biological structure separation, targeted drug delivery, and bioimaging applications. In oncological diseases treatment, the combination of magnetic nanoparticles with Au has opened novel perspectives to attain more efficient and diverse therapies for different kinds of tumors. Au MNPs have high absorption and efficiency in generating secondary electrons under g-ray or X-ray irradiation, so much interest has been raised in applying AuMNPs as radiosensitizers in RT for cancer [46]. It has been shown that Au MNPs with different sizes and shapes can significantly improve the effectiveness of cancer therapy mediated by RT and HT. Based on the efficiency of the reported results, the composition of Au–iron oxide nanocomposites is a crucial factor in regulating their physicochemical properties, conditioning their performance concerning multimodal imaging and therapy [47]. The utilization of silica (SiO_2_) as a protective coating for the magnetic core is supported by its unique characteristics, including chemical and magnetic stability, as well as its compatibility for surface functionalization. Additionally, in addition to preserving the integrity of magnetic nanoparticles, silanol groups (-SiOH) can serve as attachment sites for additional modifications [2].

### 3.2. Multifunctional Organic Material

The organic material utilized in the production of MNPs serves two primary functions: first, by incorporating organic small molecules or surfactants (e.g., sodium citrate, oleic acid, etc.) as dispersants or stabilizers during the preparation process, and second, by grafting specific binding components of the target onto the MNPs to enable precise and effective delivery akin to a key fitting into a lock. Examples of such binding components include antibodies, aptamers, lectins, and phages, among others. Detailed information on the organic molecular materials presently employed in MNPs and their respective applications can be found in Table 1.

### 3.3. Polymer Material

Polymers, such as polyethylene glycol, polyvinyl pyrrolidone, polyvinyl alcohol, poly-(lactic-co-glycolic acid), chitosan, hyaluronic acid, and polyethyleneimine, are synthetic polymer materials that possess numerous functional groups on their surface, exhibiting excellent biocompatibility. Polymer-modified MNPs can be synthesized using various methods such as physical adsorption [63], chemical coupling [64], and surface self-assembly procedures [65]. These polymers on MNPs not only facilitate effective interaction with the target substances but also prevent nanoparticles agglomeration [19]. This approach enhances synthesis stability, pharmacokinetics, and biocompatibility. In MFH, the most clinically developed MNPs are typically stabilized by biocompatible hydrophilic surface coatings such as polyethylene glycol, aminosilane, or dextran [66]. Veloso et al. made the composites through the interplay of (di)phenylalanine-coated magnetic nanoparticles, PEGylated liposomes and doxorubicin co-assembly in dehydropeptide-based gels, enabling an enhancement of the gelation kinetics in a concentration-dependent manner, mainly through the use of PEGylated liposomes. The composites can not only control the behavior of the system through the externally applied magnetic field but also achieve the biocompatibility of the particles [67]. Research has demonstrated that the surface of bacteria contains lipopolysaccharides, teichoic acids, and other components, indicating a negative charge characteristic. Therefore, MNPs modified with positively charged polymers can efficiently and non-selectively adhere to bacterial surfaces through electrostatic interactions [68].

## 4. Applications of MNPs in Biomedical Fields

### 4.1. Applications of MNPs in Medical Imaging Technology

MRI is one of the main in vivo imaging modalities, being able to provide both anatomical and functional information with excellent image quality. Superparamagnetic iron oxide nanoparticles (SPIONs) have emerged as a promising alternative to conventional contrast agents for MRI [69]. SPIONs shorten T_2_ relaxation time, decrease the signal intensity of protons, and form dark areas in MRI images to enhance contrast. SPIONs have been used for applications in the diagnostic imaging of major cancer types [70]. Recently, an ultrasmall manganese ferrite nanoparticle modified with polyethylene glycol-ethoxy-benzyl ligand on the surface (MnFe_2_O_4_-EOB-PEG) developed by Fan Haiming’s team has achieved high hepatocyte specificity that is ultrasensitive, and the detection rate of ultrasmall liver tumors by MnFe_2_O_4_-EOB-PEG was as high as 92%, which is much higher than that of clinical hepatocyte specific contrast agent Gd-EOB-DTPA (48%). It is suitable for high-resolution hepatobiliary MRI in large animals, which can quickly detect ultrasmall liver tumors below 5 mm [71]. SPIONs serve as versatile contrast agents that are capable of being used individually or in combination as bimodal imaging agents for techniques like positron emission tomography (PET) [72]. Furthermore, SPIONs can be integrated into diagnostic and therapeutic materials for applications such as magnetic hyperthermia [73]. SPIONs have been approved by the U.S. Food and Drug Administration (FDA) for clinical use: Combidex^®^ (U.S.) and Sinerem^®^ (Europe) as a magnetic resonance imaging (MRI) agent. In addition to in vivo focal tissue imaging, Ferumoxytol is currently the only MRI angiography agent used to characterize and map metastatic lymph nodes [74].

In addition to MRI, the emerging medical imaging technology currently favored by researchers is magnetic particle imaging (MPI), which has higher sensitivity and contrast. MPI generates high-resolution three-dimensional images of the concentration and location of iron oxide nanoparticle (IONP) tracers after they have been injected into the bloodstream intravenously, which relies on the change in the direct magnetization of the IONPs. Unlike MRI, which is based on changes in the nuclear magnetization of surrounding water molecules, MPI is capable of utilizing IONP tracers for real-time site and concentration detection for true quantification [30]. Early applications of MPI have focused on various applications such as MPI cell tracking, multiplex MPI, perfusion and tumor MPI, lung MPI, and functional MPI [75,76,77].

### 4.2. Applications of MNPs in the Treatment of Diseases

#### 4.2.1. Applications of MNPs in Magnetic Fluid Hyperthermia

Magnetothermal therapy is a form of physical therapy utilized in the clinical management of cancer either as a standalone treatment or in conjunction with other therapeutic approaches such as ionizing radiation therapy and chemotherapy. This therapy exploits the enhanced permeability and retention effects of tumor tissue vasculature to treatment. MFH with magnetic iron oxide nanoparticles was clinically approved in 2010 for treating recurrent glioblastoma with radiation following demonstrations of improved overall survival in clinical trials [74,78]. Issels et al. found that a combination of regional hyperthermia and neoadjuvant chemotherapy resulted in increased local progression-free survival as well as improved overall survival for patients with localized high-risk soft tissue sarcoma in a phase III randomized clinical trial (EORTC 62961-ESHO 95) [79]. Van Landeghem et al. present a study detailing the autopsy results of two glioblastoma patients who underwent treatment with MFH following the instillation of MNPs [80]. The majority of the nanoparticles were aggregated and preferentially localized in areas of geographic necrosis within the tumor, which was restricted in distribution to the sites of instillation. At the borders of the aggregates, the particles were phagocytosed mainly by macrophages. No bystander effect of the instillated nanoparticles could be observed regarding, e.g., sarcomatous tumor formation, the formation of a sterile abscess, or foreign body giant cell reaction.

Moreover, moderate hyperthermia (39–45 °C) demonstrates a promising therapeutic effect and activates local immune vitality. This phenomenon induces a cellular stress cascade, referred to as the unfolded protein response, within the tumor microenvironment, subsequently eliciting an immune response. In the study, 43 °C was able to trigger evidence of an anti-tumor CD8+ T cell response [68], whereas 45 °C did not, suggesting that a narrower target window may be required for effective immune modulation. Evidence that temperatures of 43 °C were able to elicit an anti-tumor CD8+ T cell response, whilst temperatures of 45 °C were not, in the same experimental system [81], indicate that there may be a narrow target window required for effective immunomodulation.

#### 4.2.2. Drug Delivery and Targeting

With the application of nanotechnology in biomedicine, MNP drug-carrying systems have been applied to targeted drug therapy for inflammation, infection [82], autoimmune diseases [83], and cancer [84], which involves the use of biologic drugs, the target identification of markers, and metabolic property studies [74,85]. In the process of preparing MNPs, existing drugs are predominantly loaded onto nanoparticles through various methods. The first method involves the combination of nanoparticles and drugs, facilitated by weak intermolecular interaction forces such as hydrogen bonding, hydrophobic effects, electrostatic interactions, van der Waals’ forces, and spatial interactions. The second method entails the direct bonding of particles onto drugs through surface functional groups, including hydroxyl, amino, and carboxyl groups. Drug-loaded MNPs can successfully evade in vivo barrier interception and achieve in vivo shuttle freedom by maintaining a controlled particle size of 10–100 nm and subsequently delivering the drug precisely under the influence of an applied magnetic field [86].

The ideal delivery of drug-loaded MNPs can achieve precise localization, strong targeting, and lower toxicity, making it particularly advantageous for advanced or intolerant patients who cannot undergo conventional radiotherapy or chemotherapy. Therefore, drug-carrying MNP targeted therapy represents a contemporary approach to cancer treatment that significantly prolongs the survival period and improves the quality of patients’ survival [87]. Furthermore, drug-loaded MNPs play a crucial role in identifying disease targets and investigating drug metabolism properties. Japanese scientists Takumi Ito et al. used thalidomide beads to identify the target protein of thalidomide, demonstrating that cereblon (CRBN), a protein encoded by a candidate gene associated with mild mental retardation, serves as a primary target for thalidomide teratogenicity [88]. Zeid et al. found that IONPs formed a more stable complex with the spike protein receptor binding domain (S1-RBD) of SARS-CoV-2, leading to alterations in the conformation of the envelope and spiny protein subunits of SARS-CoV-2, ultimately resulting in viral inactivation. This finding suggests a potential role for IONPs in combating novel coronavirus pneumonia [89].

### 4.3. Applications of MNPs in Biomedical Assays

MNPs can achieve the purification of various biomolecules through techniques such as magnetic separation and magnetic solid-phase extraction. This approach facilitates the separation and purification of target substances without the need for intricate chromatographic equipment. Therefore, MNPs are widely used in biomedical fields, such as nucleic acid preparation, immunoassay, cell sorting, protein purification, early diagnosis of tumor cells and biomarkers, and the isolation of cells, viruses, and exosomes [90].

The surface modification of MNPs utilized various functional groups, which can immobilize different biological ligands in the analyte [59]. Commonly used MNPs include histidine-tagged MNPs [91], diethylaminoethylcellulose MNPs [92], heparin MNPs [93], flag-labeled MNPs, antibody MNPs (Protein A/Protein G/Protein L) [94,95], Strep-tag II MNPs [96], adapter MNPs [97], and solid-phase peptide MNPs [98,99], primarily for the purpose of separating and purifying the analyte.

#### 4.3.1. Detection of Nucleic Acid

**Figure 2:**
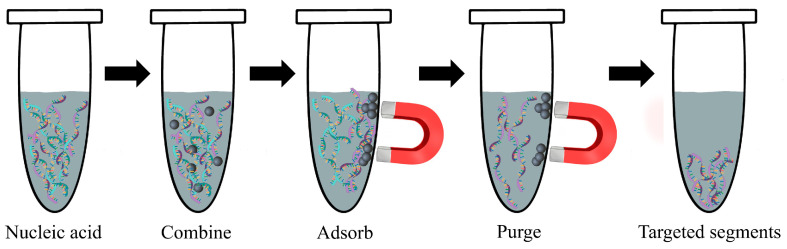
A schematic diagram depicting the process of nucleic acid molecule extraction and purification utilizing magnetic nanoparticles (MNPs). The MNPs selectively bind to the target nucleic acid fragment, facilitating enrichment and separation under the influence of a magnetic field. Subsequently, the MNPs disassociate from the nucleic acid fragment, allowing for the collection of the desired target fragment.

The use of MNPs in nucleic acid testing is mainly for the extraction and purification of nucleic acid molecules as well as the determination of specific sequences of nucleic acids (Figure 2), such as the measuring of single nucleotide polymorphisms (genetic variations) [100], real-time PCR quantification [101], multiplex PCR assays [102], forensic identification [103], and clinical diagnostics [104].

**Figure 3:**
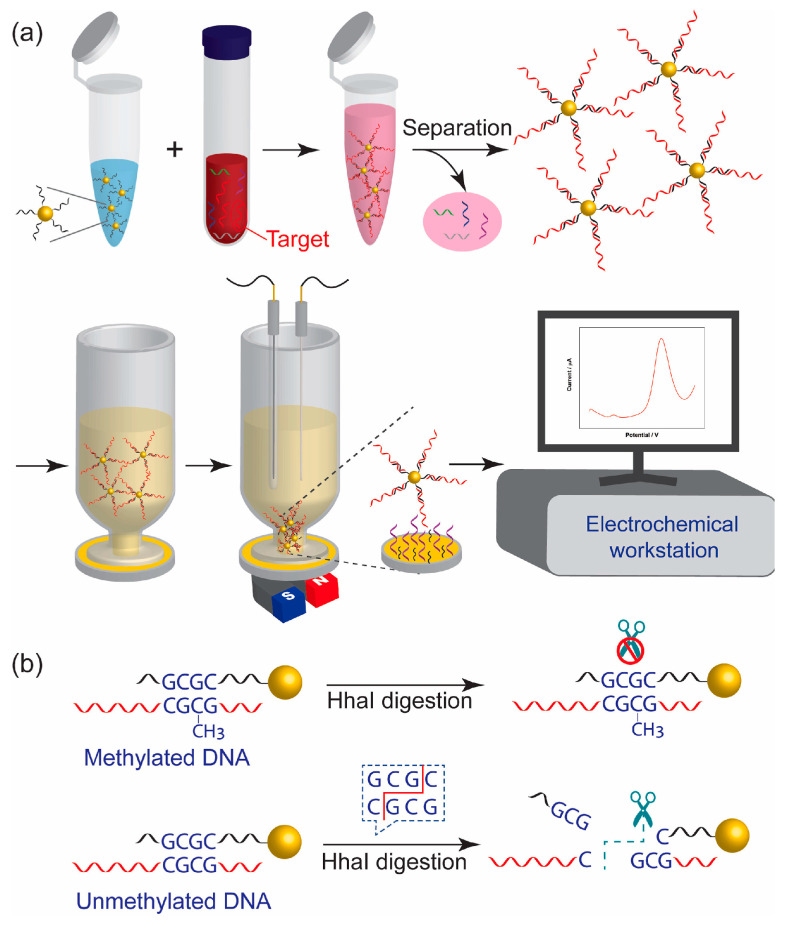
Schematic illustration of the detection of DNA methylation based on nucleic acid hybridization on a network of gold-coated magnetic nanoparticles. (**a**) Workflow for the measurement of methylated DNA. (**b**) Principle of the specific detection of DNA methylation. Methylated DNA survived HhaI digestion, whereas unmethylated DNA was digested by the HhaI restriction enzyme. The use of Hhal restriction endonuclease facilitates the selective and sensitive detection of 110 nucleotide DNA targets with a single-site 5-methylcytosine. Image used with kind permission from Biosensors and Bioelectronics (ref. [76]). Copyright 2024, Biosensors and Bioelectronics.

The automatic and rapid nucleic acid extractor (NAE) designed by Chen et al. can create 16 samples simultaneously, and the extraction process can be finished within 30 min [105]. Li et al. employed a pair of universal dual-color probes to four different SNP loci (C667T, A1298C, M235T, and G93A), thus greatly reducing typing costs [106]. Wang et al. designed a novel competitive chemiluminescent DNA method based on Fe_3_O_4_@SiO_2_@Au-functionalized magnetic nanoparticles (Au-MNPs) for the detection of the p53 tumor suppressor gene with a detection limit of 0.001 ng/mL (0.16 pM) and a wide linear response range (0.001 ng/mL~6.6 ng/mL) [107]. The DNA-Au@MNPs-based sensor developed by Chen et al. demonstrates potential for the swift identification of DNA methylation in blood, offering a response time of 35 min and enabling a minimally invasive diagnosis of ovarian cancer. The biosensor presents a dynamic range from 2 aM to 20 nM for 110 nucleotide DNA sequences containing single-site methylation with the lowest detected concentration of 2 aM [108] (Figure 3).

**Figure 4:**
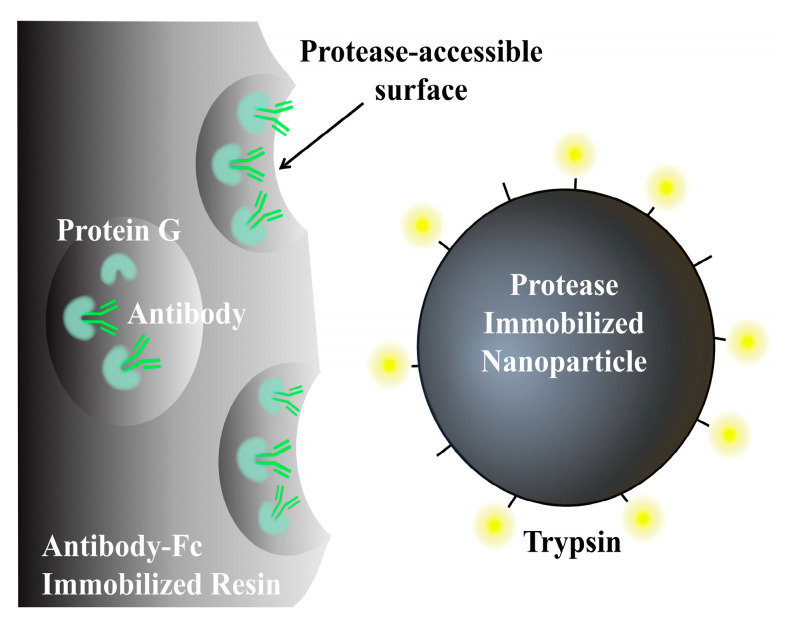
Conceptual representation of nano-surface and molecular-orientation limited (nSMOL) proteolysis. Protease is immobilized on spherical nanoparticles and antibodies on fine-pore Protein G resin. The arrowed area represents the protease-accessible surface.

At present, there is a wide array of high-throughput, high-sensitivity, and automated MNP products available in the commercial market. Commonly utilized commercial products include Primer Design from Eastleigh, UK, Magtivio from Thermiekstraat, the Netherlands, Omega Bio-Tek from Norcross, GA, USA, as well as BIOBASE (Jinan, China), MAGIC-BIO (Lishui, China), and BIO-LAB (Xi’an, Chin) from China. Additionally, a collaborative effort between a Japanese research team and Shimadzu Corporation (Kyoto, Japan) has resulted in the development of 200-nanometer-sized magnetic beads (FG beads) capable of identifying specific sequences of monoclonal antibodies for clinical monitoring of antibody–drug metabolism [109], which have been widely used in the contract research organization (CRO) of biopharmaceuticals (Figure 4).

#### 4.3.2. Cell Separation

**Figure 5:**
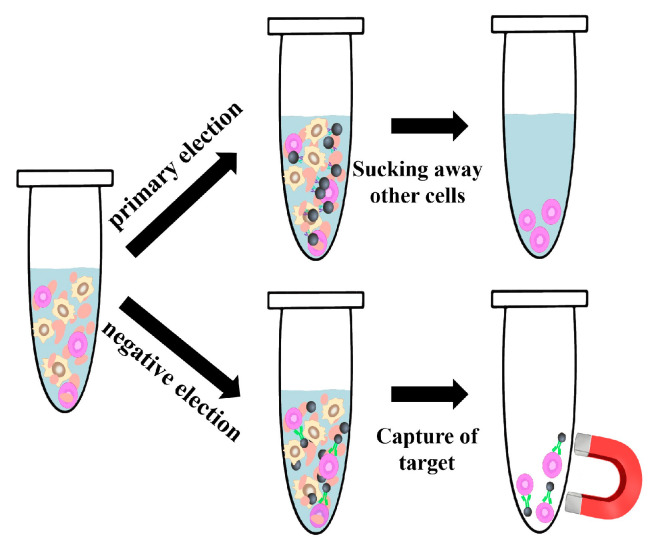
Schematic illustration of two cell separation methods for magnetic nanoparticles (MNPs). Positive selection involves enriching and purifying target cells by binding MNPs to them, while negative selection separates target cells by binding MNPs to unwanted cells.

During cell separation, MNPs are bound to specific biorecognition molecules such as antigens, glycoproteins, and specific structural components on the cell surface by Magnetic Activated Cell Sorting (MACS) [110,111,112], nucleic acid probes, antibiotics, phages [113], and lectins [114,115]. Subsequently, the MNP-labeled cells are separated from the mixtures through the application of a magnetic field, leading to the enrichment and purification of the target cells. Current MNP cell sorting techniques primarily consist of positive and negative sorting methods. Positive sorting involves MNPs binding to target cells for isolation and purification, making it suitable for flow analysis and cell-based analysis. Positive sorting can be further categorized into direct and indirect positive sorting. Indirect positive sorting involves the labeling target cells with a non-coupled, biotinylated, or fluorescein-coupled primary antibody and then attaching it to the MNPs using lgG, anti-biotin–streptavidin, or anti-fluorescein secondary antibodies to separate virtually any cell type based on the specific binding, affinity, and adsorption. In contrast, negative sorting involves MNPs binding to unwanted cells, leaving behind the target cells (Figure 5). When applying nanoscale MNPs for cell sorting, MNPs with sizes less than 250 nm are stable and biocompatible and do not affect the physiological function of cells, which has a wide range of applications in tumor research, cellular immunotherapy, and single-cell analysis [116].

At present, the FDA and the European Union have approved the operation of two cell separation systems based on immunomagnetic separation technology. The first is the CELLSEARCH system for clinical diagnoses and enumeration of circulating tumor cells (CTCs) in patients bearing metastatic breast cancer and metastatic colorectal or prostate cancer [117]. The CELLSEARCH system is a semi-automated in vitro diagnostic device that identifies, isolates, and counts CTCs from a simple blood test using a ferrofluid of IONPs modified with the anti-epithelial cell adhesion molecule antibody EpCAM. The second was Miltenyi Biote’s introduction of the CliniMACS CD34 Reagent System as the first semi-automated immunomagnetic cell separation system for clinical allogeneic stem cell transplantation and enrichment of hematopoietic stem cells expressing CD34+ in patients with acute myeloid leukemia [118].

#### 4.3.3. Determination of Other Biomolecules

Various techniques, such as magnetic solid-phase extraction [119], immobilization [120], and paramagnetic probes [121], are commonly employed for the purification and characterization of biomolecules, including proteins, pathogenic bacteria, viruses, and biomarkers. The SARS-CoV-2 aerosol detection platform developed by Chen H et al. used a wet-wall cyclone in combination with immunomagnetic nanoparticle adsorption sampling and ddPCR for the detection of airborne SARS-CoV-2 aerosols [122] with a minimum detection limit of 250 copies per unit volume of aerosol (102 copies/mL, concentration factor 2.5).

MNPs also play an indelible role in the detection of much-anticipated cancer biomarkers. Magnetoimmunoassay-based electrochemical sensors, photoelectrochemical sensors, and magnetofluidic systems are capable of rapidly and efficiently extracting and determining biomarkers in complex biological samples for in vitro detection [123,124]. Chikkaveeraiah et al. used a microfluidic system equipped with heavy enzyme-labeled MNPs to detect the cancer biomarker proteins prostate-specific antigen (PSA) and interleukin-6 (IL-6) in serum [125], and the detection limit of PSA was 0.23 pg/mL and 0.30 pg/mL for IL-6 in diluted serum mixtures.

### 4.4. Other Applications of MNPs in the Treatment of Diseases

In addition to the aforementioned biomedical applications, MNPs are extensively utilized in magnetic transfection technology, cell therapy, deep brain stimulation (DBS), and the repair and regeneration of damaged tissues and organs. Among the physical methods to modulate neuronal activity without hardware implants, only magnetic fields can penetrate the brain without absorption or scattering [126], so various methods of neuronal modulation by MNPs have been swiftly developed to improve existing therapeutic strategies and provide new therapeutic ideas [72]. The predominant magnetic DBS methods are mainly magnetothermal and magneto-mechanical stimulation. Magnetic nanostructures (MNSs) used in the fields of tissue engineering and regenerative medicine have the capability to provide magnetic targeting to stem cells, thereby facilitating cell differentiation through the mechanical stimulation induced by MNSs. Furthermore, the scaffolds created by MNSs enhance cell proliferation and adhesion, consequently enhancing the effectiveness of the scaffolds in vivo and promoting tissue repair [127]. In addition, mechanical stimulation generated by applying a magnetic field may activate some receptors on the cell surface and thus upregulate growth-related genes [128].

From the above discussion, the diverse applications of MNP product development encompass both in vivo and in vitro applications such as medical imaging, magnetic hyperthermia, targeted drug delivery, cellular therapy, DBS, nucleic acid detection, cell separation, magnetic transfection, and magnetic solid-phase extraction technologies. Among these applications, in vitro MNP products have been slowly replacing previous technologies as the mainstream choice due to their characteristic advantages and are widely used. In vivo MNP products, due to the longer clinical trial period, can be applied to a limited number of products at present, although the results are still objective.

## 5. Conclusions and Future Perspectives

Magnetic nanomaterials, as a distinctive component within the field of nanobiomaterials, have garnered significant attention from researchers and are increasingly being acknowledged for their diverse applications. The emergence of magnetic nanotechnology presents novel opportunities in the medical realm, particularly in addressing complex human diseases such as tumors and neurodegenerative disorders. The consumer preference for various magnetic nanomaterial products in the market is attributed to their convenience and eco-friendly nature, rendering them well-suited for contemporary in vitro biomedical applications. Consequently, MNPs are anticipated to play a pivotal role as the “material of the future” and will have a significant impact on all areas of nanobiomedicine.

Despite the promising results of previous studies on MNPs, there remain numerous obstacles to be addressed during the clinical trial phase. These challenges include investigating the internalization effects of MNPs, their clearance in vivo, and potential barriers such as long-term toxicity (including cytotoxicity, hematotoxicity, teratogenicity, and mutagenicity). Strategies to mitigate these issues have involved adjusting the size and functional modifications of MNPs to minimize their toxic effects and promote targeted accumulation at specific pathological sites. In vitro assay studies, the targeted adsorption of specific genera and specific microorganisms (e.g., Mycoplasma, Chlamydia, and parasites) is rarely studied [89], and the cost associated with targeting these microorganisms to address diseases and hazards remains high in terms of financial and resource allocation. Secondly, MNP detection products based on antibodies, lectins, and antibiotics account for a major share of the market. Except for antibody MNPs and nucleic acid detection MNP products, it is difficult to dissociate the target and reuse other MNP products, so many of them are disposable products, which greatly increases the cost of use and resource waste. By employing ion substitution and reversible competitive action, the substances attached to MNPs can be dissociated, enabling the reuse of MNP products. Consequently, the development of multifunctional applications utilizing MNPs is anticipated to become a common practice in industrial and biomedical markets, offering promising opportunities for future utilization.

## Figures and Tables

**Table 1:** Organic molecules modified on MNPs and their mechanism of action.

Organic Molecules	Mechanism of Action	Appliance	Reference
amino group	The positively charged surfaces of MNPs facilitate electrostatic interactions and hydrogen bonding	Attachment of groups, binding of DNA, or capture of bacteria	Bai et al. [48]
carboxyl group (-COOH)	The carboxyl group facilitates the formation of ionic bridges between sodium ions in solution and the phosphate groups of nucleic acid molecules	Linkage groups, specific adsorption of nucleic acids	Li et al. [49]
Salicylic acid (SA)	Introducing carboxylic acid and phenolic functional groups onto MNPs	Make MNPs have good adsorption properties	Zhou et al. [50]
Acridine Orange, (ACO)	ACO is a cell-permeable fluorescent and water-soluble stain, while MNPs@ACO exhibits the ability to interact with DNA and RNA through embedding or electrostatic attraction	binding nucleic acid	Sahoo et al. [51]
Imidazole (IMI)	The charge of MNPs@IMI can reach neutrality and exhibits reversible charge behavior upon pH modification	adsorbing DNA by electrostatic action	Maeda et al. [52]
agglutinin	This sugar-binding protein possesses one or more glycosyl binding sites within its three-dimensional structure, enabling it to interact with peptidoglycan and lipopolysaccharide present on the surface of diverse cell types, thereby inducing agglutination or glycoconjugate precipitation in a wide range of cellular contexts	Binding bacteria in a broad spectrum	Kaitlin et al. [53]
antibiotics	Antibiotic-modified MNPs exhibit antibacterial activity through specific binding interactions with bacterial surface structures	Specific recognition of ligands, target drugs	Abdelaziz et al. [54]
bacteriophage	The phage tail fibers exhibit a recognition and binding capability toward bacteria	Biometric ligands, specific isolation of target pathogens	Zhan et al. [55]
amino acids	Numerous side-chain amino acids possess plentiful carboxyl, hydroxyl, and sulfhydryl groups, thereby offering a significant quantity of binding sites for nanoparticles	Functionalized modification, capture of bacteria	Antal et al. [56]
polypeptides	Selective and potent signaling molecules that bind to specific cell surface receptors (e.g., G protein-coupled receptors or ion channels) to trigger intracellular effects	Specific recognition of ligands and target drugs	Kuan et al. [57]
enzymes	MNPs@enzyme serves to safeguard enzyme activity while concurrently functioning as a magnetic separation and recovery tool	Enzyme-carrier complexes with high stability and selectivity	Matveeva et al. [58]
streptomycin (antibiotic)	Streptavidin demonstrates a high degree of specificity and a robust affinity for tetrameric biotin binding	Commonly used as affinity-adsorbed MNPs for biological use	Sosa-Acosta et al. [59]
liposome	Magnetic-fluid-loaded liposomes (MFLs) possess a positively charged surface that enables them to interact with phosphorylates in DNA. MFLs have the ability to adsorb to cell membranes, which are negatively charged, and subsequently enter the cell through membrane depressions, thereby leveraging the benefits of both magnetic materials and liposomes	Carriers of targeted drugs	Millart et al. [60]
antibodies	Antibody-modified MNPs, commonly referred to as immunomagnetic beads, exhibit specific binding capabilities to antigens	Specific binding ligands and targeting drugs	Liu et al. [61]
aptamers	1. The recognition of ligands occurs through the mutual alignment of spatial conformations, resulting in high selectivity and affinity for their respective targets. 2. The termini of aptamer sequences may be adorned with a variety of functional groups or molecules to facilitate chemical modification and sensing, including but not limited to amino, carboxyl, biotin, and fluorescein	Acting as affinity adsorption and specific binding, applying in the fields of magnetic transfection and gene therapy	Sizikov et al. [62]

## Data Availability

Not applicable.

## References

[1] Kianfar E. (2021). Magnetic nanoparticles in targeted drug delivery: A review. J. Supercond. Nov. Magn..

[2] Hosu O., Tertis M., Cristea C. (2019). Implication of Magnetic Nanoparticles in Cancer Detection, Screening and Treatment. Magnetochemistry.

[3] Gessner I., Park J.H., Lin H.Y. (2022). Magnetic gold nanoparticles with idealized coating for enhanced point-of-care sensing. Adv. Healthc. Mater..

[4] Daya R., Xu C., Nguyen N.Y.T. (2022). Angiogenic hyaluronic acid hydrogels with curcumin-coated magnetic nanoparticles for tissue repair. ACS Appl. Mater. Interfaces.

[5] Katz E. (2020). Magnetic nanoparticles. Magnetochemistry.

[6] Zhang Y., Zhang Q., Zhang A. (2019). Multifunctional co-loaded magnetic nanocapsules for enhancing targeted MR imaging and in vivo photodynamic therapy. Nanomed. Nanotechnol. Biol. Med..

[7] Yang Y., Li T., Jing W. (2023). Dual-modality and Noninvasive Diagnostic of MNP–PEG–Mn Nanoprobe for Renal Fibrosis Based on Photoacoustic and Magnetic Resonance Imaging. ACS Appl. Mater. Interfaces.

[8] Ma R., Hou Y., Wang S. (2023). Imaging-Guided Cancer Therapy Based on Multifunctional Magnetic Nanoparticles. Chin. J. Chem..

[9] Wong J., Prout J., Seifalian A. (2017). Magnetic nanoparticles: New perspectives in drug delivery. Curr. Pharm. Des..

[10] Wang N., Guan Y., Yang L. (2013). Magnetic nanoparticles (MNPs) covalently coated by PEO–PPO–PEO block copolymer for drug delivery. J. Colloid Interface Sci..

[11] Demessie A.A., Park Y., Singh P. (2022). An Advanced Thermal Decomposition Method to Produce Magnetic Nanoparticles with Ultrahigh Heating Efficiency for Systemic Magnetic Hyperthermia. Small Methods.

[12] Kaushik S., Thomas J., Panwar V.A. (2022). drug-free strategy to combat bacterial infections with magnetic nanoparticles biosynthesized in bacterial pathogens. Nanoscale.

[13] Willis A.J., Pernal S.P., Gaertner Z.A. (2020). Rotating magnetic nanoparticle clusters as microdevices for drug delivery. Int. J. Nanomed..

[14] Ni D., Ferreira C.A., Barnhart T.E. (2018). Magnetic targeting of nanotheranostics enhances cerenkov radiation-induced photodynamic therapy. J. Am. Chem. Soc..

[15] Ostroverkhov P., Semkina A., Naumenko V. (2018). HSA—Coated magnetic nanoparticles for MRI-guided photodynamic cancer therapy. Pharmaceutics.

[16] Chen C., Wang S., Li L. (2016). Bacterial magnetic nanoparticles for photothermal therapy of cancer under the guidance of MRI. Biomaterials.

[17] Yeboah I.B., Hatekah S.W., Yaya A. (2022). Photothermally-heated superparamagnetic polymeric nanocomposite implants for interstitial thermotherapy. Nanomaterials.

[18] Kang S., Baskaran R., Ozlu B. (2020). T1-positive Mn2+-doped multi-stimuli responsive poly (L-DOPA) nanoparticles for photothermal and photodynamic combination cancer therapy. Biomedicines.

[19] Xiao F., Li W., Xu H. (2022). Advances in magnetic nanoparticles for the separation of foodborne pathogens: Recognition, separation strategy, and application. Compr. Rev. Food Sci. Food Saf..

[20] Azadpour B., Aharipour N., Paryab A. (2023). Magnetically-assisted viral transduction (magnetofection) medical applications: An update. Biomater. Adv..

[21] Guo Y., Li S., Wang Y. (2017). Diagnosis–Therapy integrative systems based on magnetic RNA nanoflowers for Co-drug delivery and targeted therapy. Anal. Chem..

[22] Farooq A., Tosheva L., Azzawi M. (2016). Real-time observation of aortic vessel dilation through delivery of sodium nitroprusside via slow release mesoporous nanoparticles. J. Colloid Interface Sci..

[23] Liu H., Li S., Wang Z. (2008). Automated detection system of single nucleotide polymorphisms using two kinds of functional magnetic nanoparticles. Appl. Surf. Sci..

[24] Zhang J., Zhang T., Gao J. (2022). Biocompatible iron oxide nanoparticles for targeted cancer gene therapy: A review. Nanomaterials.

[25] Brestovac B., Harnett G.B., Smith D.W. (2005). Multiplex nested PCR (MNP) assay for the detection of 15 high risk genotypes of human papillomavirus. J. Clin. Virol..

[26] Jun Y., Seo J., Cheon J. (2008). Nanoscaling laws of magnetic nanoparticles and their applicabilities in biomedical sciences. Acc. Chem. Res..

[27] Bai S., Hou S., Chen T. (2024). Magnetic nanoparticle-mediated hyperthermia: From heating mechanisms to cancer theranostics. Innov. Mater..

[28] Fallows T.W., McGrath A.J., Silva J. (2019). High-throughput chemical and chemoenzymatic approaches to saccharide-coated magnetic nanoparticles for MRI. Nanoscale Adv..

[29] Quan K., Zhang Z., Ren Y. (2021). Possibilities and impossibilities of magnetic nanoparticle use in the control of infectious biofilms. J. Mater. Sci. Technol..

[30] Bruschi M.L., de Toledo L.A.S. (2019). Pharmaceutical applications of iron-oxide magnetic nanoparticles. Magnetochemistry.

[31] Obaidat I.M., Narayanaswamy V., Alaabed S. (2019). Principles of magnetic hyperthermia: A focus on using multifunctional hybrid magnetic nanoparticles. Magnetochemistry.

[32] Rezaei B., Yari P., Sanders S.M. (2024). Magnetic nanynchronous disintegration of ferroptosis defense axis via engineered exosome-conjugated magnetic nanopaapplications. Small.

[33] Arias L.S., Pessan J.P., Vieira A.P.M. (2018). Iron Oxide Nanoparticles for Biomedical Applications: A Perspective on Synthesis, Drugs, Antimicrobial Activity, and Toxicity. Antibiotics.

[34] Li B., Chen X., Qiu W. (2022). Synchronous disintegration of ferroptosis defense axis via engineered exosome-conjugated magnetic nanoparticles for glioblastoma therapy. Adv. Sci..

[35] Bilal M., Mehmood S., Rasheed T. (2019). Bio-catalysis and biomedical perspectives of magnetic nanoparticles as versatile carriers. Magnetochemistry.

[36] Slimani Y., Hannachi E., Tombuloglu H. (2020). Magnetic nanoparticles based nanocontainers for biomedical application. Smart Nanocontainers.

[37] Bao S., Yang W., Wang Y. (2020). One-pot synthesis of magnetic graphene oxide composites as an efficient and recoverable adsorbent for Cd (II) and Pb (II) removal from aqueous solution. J. Hazard. Mater..

[38] Tran H.V., Ngo N.M., Medhi R. (2022). Multifunctional Iron Oxide Magnetic Nanoparticles for Biomedical Applications: A Review. Materials.

[39] Abd Elrahman A.A., Mansour F.R. (2019). Targeted magnetic iron oxide nanoparticles: Preparation, functionalization and biomedical application. J. Drug Deliv. Sci. Technol..

[40] Dulińska-Litewka J., Łazarczyk A., Hałubiec P. (2019). Superparamagnetic Iron Oxide Nanoparticles—Current and Prospective Medical Applications. Materials.

[41] Hepel M. (2020). Magnetic Nanoparticles for Nanomedicine. Magnetochemistry.

[42] Sabale S., Kandesar P., Jadhav V. (2017). Recent developments in the syn-thesis, properties, and biomedical applications of core/shell superparamagnetic iron oxide nanoparticles with gold. Biomater. Sci..

[43] Zhou X., Li P., Wu X. (2022). Multifunctional biosensor constructed by Ag-coating magnetic-assisted unique urchin core porous shell structure for dual SERS enhancement, enrichment, and quantitative detection of multi-components inflammatory markers. Biosens. Bioelectron..

[44] Alyassin Y., Sayed E.G., Mehta P. (2020). Application of mesoporous silica nanoparticles as drug delivery carriers for chemotherapeutic agents. Drug Discov. Today.

[45] Bai Y.L., Shahed-Al-Mahmud M., Selvaprakash K. (2019). Tail fiber protein-immobilized magnetic nanoparticle-based affinity approaches for detection of Acinetobacter baumannii. Anal. Chem..

[46] Montiel Schneider M.G., Martín M.J., Otarola J. (2022). Bio-medical Applications of Iron Oxide Nanoparticles: Current Insights Progress and Perspectives. Pharmaceutics.

[47] Yang D., Yang G., Yang P. (2017). Assembly of Au Plasmonic Photothermal Agent and Iron Oxide Nanoparticles on Ultrathin Black Phosphorus for Targeted Photothermal and Photodynamic Cancer Therapy. Adv. Funct. Mater..

[48] Bai Y., Roncancio D., Suo Y. (2019). A method based on amino-modified magnetic nanoparticles to extract DNA for PCR-based analysis. Colloids Surf. B Biointerfaces.

[49] Li P., Li M., Zhang F. (2021). High-efficient nucleic acid separation from animal tissue samples via surface modified magnetic nanoparticles. Sep. Purif. Technol..

[50] Zhou Z., Kadam U.S., Irudayaraj J. (2013). One-stop genomic DNA extraction by salicylic acid-coated magnetic nanoparticles. Anal. Biochem..

[51] Sahoo S.L., Liu C.H. (2015). Adsorption behaviors of DNA by modified magnetic nanoparticles: Effect of spacer and salt. Colloids Surf. A Physicochem. Eng. Asp..

[52] Maeda Y., Toyoda T., Mogi T. (2016). DNA recovery from a single bacterial nanoparticles. Colloids Surf. B Biointerfaces.

[53] Hoyt K., Barr J.R., Kalb S.R. (2021). Detection of ricin activity and structure by using novel galactose-terminated magnetic bead extraction coupled with mass spectrometric detection. Anal. Biochem..

[54] Abdelaziz M.M., Hefnawy A., Anter A. (2022). Respirable spray dried vancomycin coated magnetic nanoparticles for localized lung delivery. Int. J. Pharm..

[55] Zhan S., Fang H., Chen Q. (2022). M13 bacteriophage as biometric component for orderly assembly of dynamic light scattering immunosensor. Biosens. Bioelectron..

[56] Antal I., Strbak O., Khmara I. (2020). MRI Relaxivity Changes of the Magnetic Nanoparticles Induced by Different Amino Acid Coatings. Nanomaterials.

[57] Kuan W.C., Lai J.W., Lee W.C. (2021). Covalent binding of glutathione on magnetic nanoparticles: Application for immobilizing small fragment ubiquitin-like-specific protease 1. Enzym. Microb. Technol..

[58] Matveeva V.G., Bronstein L.M. (2021). Magnetic Nanoparticle-Containing Supports as Carriers of Immobilized Enzymes: Key Factors Influencing the Biocatalyst Performance. Nanomaterials.

[59] Sosa-Acosta J.R., Iriarte-Mesa C., Ortega G.A. (2020). DNA–Iron Oxide Nanoparticles Conjugates: Functional Magnetic Nanoplatforms in Biomedical Applications. Top. Curr. Chem..

[60] Millart E., Lesieur S., Faivre V. (2018). Superparamagnetic lipid-based hybrid nanosystems for drug delivery. Expert Opin. Drug Deliv..

[61] Liu S., Chen X., Bao L. (2020). Treatment of infarcted heart tissue via the capture and local delivery of circulating exosomes through antibody-conjugated magnetic nanoparticles. Nat. Biomed. Eng..

[62] Sizikov A.A., Kharlamova M.V., Nikitin M.P. (2021). Nonviral locally injected magnetic vectors for in vivo gene delivery: A review of studies on magnetofection. Nanomaterials.

[63] You S.M., Jeong K.B., Luo K. (2021). based colorimetric detection of pathogenic bacteria in food through magnetic separation and enzyme-mediated signal amplification on paper disc. Anal. Chim. Acta.

[64] Deng M., Wang Y., Chen G. (2021). Poly-l-lysine-functionalized magnetic beads combined with polymerase chain reaction for the detection of *Staphylococcus aureus* and Escherichia coli O157: H7 in milk. Dairy Sci.

[65] Gao X., Yao X., Zhong Z. (2018). Rapid and sensitive detection of *Staphylococcus aureus* assisted by polydopamine modified magnetic nanoparticles. Talanta.

[66] Carter T.J., Agliardi G., Lin F.Y. (2021). Potential of magnetic hyperthermia to stimulate localized immune activation. Small.

[67] Veloso S.R., Tiryaki E., Spuch C. (2022). Tuning the drug multimodal release through a co-assembly strategy based on magnetic gels. Nanoscale.

[68] Cremin K., Jones B.A., Teahan J. (2020). Scanning ion conductance microscopy reveals differences in the ionic environments of gram-positive and negative bacteria. Anal. Chem..

[69] Etemadi H., Buchanan J.K., Kandile N.G. (2021). Iron oxide nanoparticles: Physicochemical characteristics and historical developments to commercialization for potential technological applications. ACS Biomater. Sci. Eng..

[70] Bakhtiary Z., Saei A.A., Hajipour M.J. (2016). Targeted superparamagnetic iron oxide nanoparticles for early detection of cancer: Possibilities and challenges. Nanomed. Nanotechnol. Biol. Med..

[71] Zhang H., Guo Y., Jiao J. (2023). A hepatocyte-targeting nanoparticle for enhanced hepatobiliary magnetic resonance imaging. Nat. Biomed. Eng..

[72] Thomas G., Boudon J., Maurizi L. (2019). Innovative magnetic nanoparticles for PET/MRI bimodal imaging. ACS Omega.

[73] Myrovali E., Maniotis N., Samaras T. (2020). Spatial focusing of magnetic particle hyperthermia. Nanoscale Adv..

[74] Neuwelt A., Sidhu N., Hu C.A.A. (2015). Iron-based superparamagnetic nanoparticle contrast agents for MRI of infection and inflammation. Am. J. Roentgenol..

[75] Bulte J.W. (2019). Superparamagnetic iron oxides as MPI tracers: A primer and review of early applications. Adv. Drug Deliv. Rev..

[76] Ling W., Wang M., Xiong C. (2019). Synthesis, surface modification, and applications of magnetic iron oxide nanoparticles. J. Mater. Res..

[77] Healy S., Bakuzis A.F., Goodwill P.W. (2022). Clinical magnetic hyperthermia requires integrated magnetic particle imaging. Wiley Interdiscip. Rev. Nanomed Nanobiotechnol..

[78] Marchal S., Hor A.E., Millard M. (2015). Anticancer drug delivery: An update on clinically applied nanotherapeutics. Drugs.

[79] Issels R.D., Lindner L.H., Verweij J. (2018). Effect of neoadjuvant chemotherapy plus regional hyperthermia on long-term outcomes among patients with localized high-risk soft tissue sarcoma: The EORTC 62961-ESHO 95 randomized clinical trial. JAMA Oncol..

[80] van Landeghem F.K., Maier-Hauff K., Jordan A. (2009). Post-mortem studies in glioblastoma patients treated with thermotherapy using magnetic nanoparticles. Biomaterials.

[81] Toraya-Brown S., Sheen M.R., Zhang P. (2014). Local hyperthermia treatment of tumors induces CD8+ T cell-mediated resistance against distal and secondary tumors. Nanomed. Nanotechnol. Biol. Med..

[82] Wang J., Wang L., Pan J. (2021). Magneto-based synergetic therapy for im-plant-associated infections via biofilm disruption and innate immunity regulation. Adv. Sci..

[83] Atluri V.S.R., Jayant R.D., Pilakka-Kanthikeel S. (2016). Development of TIMP1 magnetic nanoformulation for regulation of synaptic plasticity in HIV-1 infection. Int. J. Nanomed..

[84] Bui M.P., Le T.A., Yoon J. (2020). A magnetic particle imaging-based navigation platform for magnetic nano-particles using interactive manipulation of a virtual field free point to ensure targeted drug delivery. IEEE Trans. Ind. Electron..

[85] Gul S., Khan S.B., Rehman I.U. (2019). A comprehensive review of magnetic nanomaterials modern day theranostics. Front. Mater..

[86] Liu Y., Cao F., Sun B. (2021). Magnetic nanoparticles: A new diagnostic and treatment platform for rheumatoid arthritis. J. Leucoc. Biol..

[87] Liu X., Zhang Y., Wang Y. (2020). Comprehensive understanding of magnetic hyperthermia for improving antitumor therapeutic efficacy. Theranostics.

[88] Ito T., Ando H., Suzuki T. (2010). Identification of a primary target of thalidomide teratogenicity. Science.

[89] Abo-Zeid Y., Ismail N.S.M., McLean G.R. (2020). A molecular docking study repurposes FDA approved iron oxide nanoparticles to treat and control COVID-19 infection. Eur. J. Pharm. Sci..

[90] Ali A., Shah T., Ullah R. (2021). Review on recent progress in magnetic nanoparticles: Synthesis, characterization, and diverse applications. Front. Chem..

[91] Han X., Cao M., Zhou B., Yu C. (2022). Specifically immobilizing His-tagged allergens to magnetic nanoparticles for fast and quantitative detection of allergen-specific IgE in serum samples. Talanta.

[92] Ajibade P.A., Paca A.M. (2019). Tris (dithiocarbamato) iron (III) complexes as precursors for iron sulfide nano-crystals and iron sulfide-hydroxyethyl cellulose composites. J. Sulfur Chem..

[93] Pires F., Silva J.C., Ferreira F.C. (2024). Heparinized Acellular Hydrogels for Magnetically In-duced Wound Healing Applications. ACS Appl. Mater. Interfaces.

[94] Vu T.T., Zhang A., Wang R. (2024). Using single antigen specificity magnetic beads for the isolation of specific antibodies against HLA antigens. HLA.

[95] Källsten M., Ghorasaini M., Hartmann R. (2020). Magnetic beads for desalting of monoclonal antibodies and antibody–drug Conjugates. Anal. Chem..

[96] Guo X., Hu F., Zhao S. (2023). Immunomagnetic separation method integrated with the Strep-Tag II system for rapid enrichment and mild release of exosomes. Anal. Chem..

[97] Zhang M., Wang Y., Wu P. (2020). Development of a highly sensitive detection method for TTX based on a magnetic bead-aptamer competition system under triple cycle amplification. Anal. Chim. Acta.

[98] Khadsai S., Seeja N., Rutnakornpituk M. (2021). Selective enrichment of zein gene of maize from cereal products using magnetic support having pyrrolidinyl peptide nucleic acid probe. Food Chem..

[99] Nan K., He M., Chen B. (2024). Histidine tag modified magnetic beads for analysis of arsenic binding proteins. Anal. Chim. Acta.

[100] Lee M.H., Leu C.C., Lin C.C. (2019). Gold-decorated magnetic nanoparticles modified with hairpin-shaped DNA for fluorometric discrimination of single-base mismatch DNA. Microchim. Acta.

[101] Chen Y., Liu Y., Shi Y. (2020). Magnetic particles for integrated nucleic acid purification, amplification and detection without pipetting. TrAC Trends Anal. Chem..

[102] Xuhong Y., Sinong Z., Jianping L. (2019). A PCR-lateral flow assay system based on gold magnetic nanoparticles for CYP2C19 genotyping and its clinical applications. Artif. Cells Nanomed. Biotechnol..

[103] Nadar S.S., Kelkar R.K., Pise P.V. (2021). The un-tapped potential of magnetic nanoparticles for forensic investigations: A comprehensive review. Talanta.

[104] Cheng H., Liu J., Ma W. (2018). Low background cascade signal amplification electrochemical sensing platform for tumor-related mRNA quantification by target-activated hybridization chain reaction and electroactive cargo release. Anal. Chem..

[105] Chen Z., Wu Y., Chen H. (2016). Design and application of automatic and rapid nucleic acid extractor using magnetic nanoparticles. J. Nanosci. Nanotechnol..

[106] Li S., Liu H., Jia Y. (2013). An automatic high-throughput single nucleotide polymorphism genotyping approach based on universal tagged arrays and magnetic nanoparticles. J. Biomed. Nanotechnol..

[107] Wang L., Yao M., Fang X. (2019). Novel competitive chemiluminescence DNA assay based on Fe_3_O_4_@ SiO_2_@Au-functionalized magnetic nanoparticles for sensitive detection of p53 tumor suppressor gene. Appl. Biochem. Biotechnol..

[108] Chen D., Wu Y., Tilley R.D. (2022). Rapid and ultrasensitive electrochemical detection of DNA methylation for ovarian cancer diagnosis. Biosens. Bioelectron..

[109] Iwamoto N., Shimada T., Umino Y. (2014). Selective detection of complementarity-determining regions of monoclonal antibody by limiting protease access to the substrate: Nano-surface and molecular-orientation limited proteolysis. Analyst.

[110] Oh S., Jung S.H., Seo H. (2018). Magnetic activated cell sorting (MACS) pipette tip for immunomagnetic bacteria separation. Sens. Actuators B Chem..

[111] González-Ravina C., Castro A.P., Palomino M.C. (2021). P–081 Microfluidic sorting does not improve clinical outcomes compared to magnetic activated cell sorting (MACS) in Assisted Re-production. Hum. Reprod..

[112] Adams J.D., Kim U., Soh H.T. (2008). Multitarget magnetic activated cell sorter. Proc. Natl. Acad. Sci. USA.

[113] Wang L., Lin H., Zhang J. (2022). Phage long tail fiber protein-immobilized magnetic nanoparticles for rapid and ultrasensitive detection of Salmonella. Talanta.

[114] Monje P.V. (2023). Human Schwann Cells in vitro II. Passaging, Purification, Banking, and Labeling of Established Cultures. Bio-Protocol.

[115] Yang G., Huang M., Wang Y. (2019). Streptavidin-exposed magnetic nanoparticles for lectin magnetic separation (LMS) of *Staphylococcus aureus* prior to three quantification strategies. Microchim. Acta.

[116] Lin Z., Lin S.Y., Xie P. (2020). Rapid Assessment of Surface Markers on Cancer Cells Using Immuno-Magnetic Separation and Multi-frequency Impedance Cytometry for Targeted Therapy. Sci. Rep..

[117] Wang L., Balasubramanian P., Chen A.P. (2016). Promise and limits of the CellSearch platform for evaluating pharmacodynamics in circulating tumor cells. Semin. Oncol..

[118] FDA Approves CliniMACS CD34 Reagent System for the Prevention of Graft-vs-Host Disease in AML. https://www.ascopost.com/issues/february-15-2014/fda-approves-clinimacs-cd34-reagent-system-for-the-prevention-of-graft-vs-host-disease-in-aml/.

[119] Manousi N., Rosenberg E., Deliyanni E. (2020). Magnetic solid-phase extraction of organic compounds based on graphene oxide nanocomposites. Molecules.

[120] Mylkie K., Nowak P., Rybczynski P. (2021). Polymer-Coated Magnetite Nanoparticles for Protein Immobilization. Materials.

[121] Miao Q., Nitsche C., Orton H. (2022). Paramagnetic Chemical Probes for Studying Biological Macromolecules. Chem. Rev..

[122] Chen H., Ma X., Zhang X. (2023). Novel aerosol detection platform for SARS-CoV-2: Based on specific magnetic nanoparticles adsorption sampling and digital droplet PCR detection. Chin. Chem. Lett..

[123] Kavetskyy T., Alipour M., Smutok O. (2021). Magneto-immunoassay of cancer biomarkers: Recent progress and challenges in biomedical analysis. Microchem. J..

[124] Gheorghiu E. (2021). Detection of pathogenic bacteria by magneto-immunoassays: A review. J. Biomed. Res..

[125] Chikkaveeraiah B.V., Mani V., Patel V. (2011). Microfluidic electrochemical immunoarray for ultrasensitive detection of two cancer biomarker proteins in serum. Biosens. Bioelectron..

[126] Chen R., Canales A., Anikeeva P. (2017). Neural recording and modulation technologies. Nat. Rev. Mater..

[127] Ovejero J.G., Spizzo F., Morales M.P. (2021). Nanoparticles for Magnetic Heating: When Two (or More) Is Better Than One. Materials.

[128] Van de Walle A., Perez J.E., Abou-Hassan A. (2020). Magnetic nanoparticles in regenerative medicine: What of their fate and impact in stem cells?. Mater. Today Nano.

